# Anosognosia in Early- and Late-Onset Dementia and Its Association With Neuropsychiatric Symptoms

**DOI:** 10.3389/fpsyt.2021.658934

**Published:** 2021-05-13

**Authors:** Manuela Tondelli, Chiara Galli, Giulia Vinceti, Luigi Fiondella, Simone Salemme, Chiara Carbone, Maria Angela Molinari, Annalisa Chiari, Giovanna Zamboni

**Affiliations:** ^1^Department of Biomedical, Metabolic, and Neural Science, University of Modena and Reggio Emilia, Modena, Italy; ^2^Primary Care Department, Azienda Unitá Sanitaria Locale di Modena, Modena, Italy; ^3^Center for Neurosciences and Neurotechnology, Università di Modena e Reggio Emilia, Modena, Italy; ^4^Neurology Unit, Baggiovara Hospital, Azienda Ospedaliero Universitaria di Modena, Modena, Italy; ^5^Nuffield Department of Clinical Neurosciences, University of Oxford, Oxford, United Kingdom

**Keywords:** anosognosia, early onset dementia, late onset dementia, neuropsychiatric symptoms, apathy, Alzheimer's disease, frontotemporal dementia

## Abstract

**Background:** The symptom anosognosia or unawareness of disease in dementia has mainly been studied in patients with late-onset dementia (LOD, ≥65 years), whereas little is known on whether it is also present in patients with early-onset dementia (EOD, <65 years). We aimed at investigating differences in anosognosia between LOD and EOD, by also studying its association with different clinical variants of EOD and the presence of neuropsychiatric symptoms.

**Methods:** A total of 148 patients, 91 EOD and 57 LOD, were recruited and underwent extended clinical assessment and caregiver interview that included questionnaires aimed at measuring anosognosia and neuropsychiatric symptoms. Differences in anosognosia between EOD and LOD and between subgroups with different clinical variants were investigated, as well as correlation between anosognosia and neuropsychiatric symptoms. A regression analysis was applied to explore the association between anosognosia and development of neuropsychiatric symptoms during disease progression.

**Results:** Median levels of anosognosia were not significantly different between EOD and LOD. Anosognosia increased overtime with disease progression and was higher in frontotemporal dementia patients or, more precisely, in frontotemporal dementia and Alzheimer's disease variants associated with involvement of the frontal lobes. Higher levels of early anosognosia were associated with higher frequency and severity of subsequent neuropsychiatric symptoms, in particular apathy, later in the course of the disease.

**Conclusion:** Anosognosia is a frequent symptom of EOD, occurring in 94.5% of all-cause EOD, and it is associated with higher risk of developing neuropsychiatric symptoms during disease progression. Recognising anosognosia may be helpful for clinicians and families to reduce diagnostic delay and improve disease managment.

## Introduction

Patients with dementia frequently are not aware of their cognitive difficulties, a neurological symptom indicated as anosognosia. Studies have reported that about 10% of patients with mild dementia have anosognosia and that this proportion increases up to 80% in patients with severe dementia (1–4), suggesting an association of anosognosia with dementia severity, as also shown by longitudinal studies showing that anosognosia increases with the progression of dementia (5, 6). In addition, anosognosia is often associated with the presence of neuropsychiatric symptoms (7, 8).

Research into anosognosia has mainly focused in dementia in old age [late-onset dementia, ≥65 years, (LOD)]. However, anosognosia in early-onset dementia (EOD, <65 years) may potentially have even more detrimental effects on patients and their families, as these people are more likely to still have working, parental, and social duties and lack of awareness of disease may expose them to diagnostic delay, reduced compliance with treatments, and even risky behaviors. Only few studies have investigated anosognosia in EOD in comparison with LOD, but their results are conflicting. One first study focusing on early- vs. late-onset Alzheimer's disease (AD) found that AD-EOD were more aware of their cognitive disturbances (i.e., had less anosognosia) compared to AD-LOD (9); on the contrary, Dourado et al. did not find significant differences between these two groups (10). The only study that included patients with clinical diagnosis other than AD [more precisely vascular dementia (VaD)] found that EOD patients were more aware of their cognitive disturbances but had greater functional impairment than LOD patients (11).

The aim of the present study was to investigate the presence and degree of anosognosia in groups of patients with EOD and LOD, including different clinical presentations, by also studying its association with neuropsychiatric symptoms and dementia progression overtime. We hypothesized that anosognosia increases along disease progression and that it might be associated with development of neuropsychiatric symptoms during the course of disease.

We anticipated that improving the quantification and characterization of anosognosia in EOD would help clinicians, patients, and their family to reduce diagnostic delay and improve identification of people at greater risk of potentially dangerous behaviors.

## Materials and Methods

Patients for this study were recruited from a larger database established for an epidemiologic study in the province of Modena, Northern Italy, including prospectively collected cases from 2017 to 2019 (12). Inclusion criteria were as follows: a dementia diagnosis with symptoms onset before age 65 (EOD) or at/after age 65 (LOD), dementia as the principal cause of disability, having a caregiver available for interview, and being a resident in the province of Modena. We compared EOD patients available in our dataset with a cohort of LOD patients seen in the same clinic without any speculation about their epidemiology or diagnosis frequencies. For the present study only, additional inclusion criteria were also the availability of anosognosia measured when patients had been first seen in the Modena dementia service (early anosognosia) and at the moment of recruitment (current anosognosia). Exclusion criteria included coexisting diagnoses of pervasive developmental disorder or major psychiatric disorder, and cognitive impairment in the context of another neurological disorder in which disability was primarily related to non-cognitive symptoms (e.g., multiple sclerosis, cerebrovascular disease with severe motor disability). For each patient, we collected demographic data, clinical information such as type of dementia diagnosis at the moment of recruitment and duration of disease from symptoms onset until recruitment, neuropsychological measures including the MMSE (13) at the moment of recruitment (current MMSE) and when patients had been first seen in the Modena dementia service (early MMSE), and measurement of anosognosia at the moment of recruitment (current anosognosia) and when patients had been first seen in the Modena dementia service (early anosognosia). Anosognosia was assessed with Clinical Insight Rating Scale (CIRS) (14). CIRS evaluation is based on the examiner's judgement about patient's level of insight based on history, neuropsychological testing, or a combination of them. It defines four domains of patient's awareness: (a) the reason for the visit; (b) the cognitive deficits; (c) functional deficits; and (d) perception of the progression of deficits; based on a separate interview with the patient and the caregiver, the items are rated by the examiner as 0 (full insight), or 1 (partial insight), or 2 (not insight), and summed to obtain a total score between 0 and 8. In addition, at the moment of recruitment, additional questionnaires including the Neuropsychiatry Inventory (NPI) (15) were administered to obtain a measurement of behavioral and psychological symptom (current NPI). Twelve neuropsychiatric symptoms were assessed (delusion, hallucination, agitation, dysphoria, anxiety, euphoria, apathy, disinhibition, irritability, aberrant motor behavior, night-time behavioral disturbances, and changes in appetite/eating behaviors).

The study was approved by local Ethical Committee; it was conducted according to the Declaration of Helsinki, and patients signed informed consent (Study Number 186/2016 of the local ethical committee).

Patients were classified according to the clinical diagnosis at the moment of recruitment in AD (16) [including amnestic variant, logopenic variant (17), posterior cortical atrophy (18) and behavioral/disexecutive variant (19)], VaD (20, 21), possible or probable dementia with Lewy body (LBD) (22), frontotemporal dementia (including behavioral variant, semantic variant of primary progressive aphasia, and non-fluent agrammatic variant of primary progressive aphasia) (17, 23), atypical parkinsonism [i.e., progressive sopranuclear palsy (PSP) or corticobasal degeneration (CBD)] (24).

Descriptive statistics were applied to investigate early and current degree of anosognosia in EOD and LOD patients, as well clinical and global cognitive measures. Differences between the two groups were investigated with parametric or non-parametric tests as appropriate; *t*-tests for independent variables or the Mann–Whitney test was applied for continuous variables, and chi-squared test was applied for binary variables. Wilcoxon signed-rank test was used to investigate differences in cognitive measures and anosognosia measures between the first visit and time of recruitment. Multivariable analysis for repeated measures was applied to examine anosognosia differences in EOD and LOD at first visit and recruitment. Univariable analysis was applied to investigate anosognosia differences in diagnostic subgroups of EOD and LOD; Bonferroni *post hoc* correction was applied for diagnostic group variable. The Spearman partial correlation was used to investigate the associations between early and current anosognosia and NPI in both groups. For NPI correlation, given to multiple correlation investigated (12 items), we considered significant a threshold of *p* < 0.004. Linear regression model was applied to investigate association between early anosognosia and presence of neuropsychiatric symptoms at recruitment assessed with NPI both in the whole cohort and in EOD and LOD separately. All statistical analyses were performed with SPSS software for Windows.

## Results

### Demographical and Clinical Features

**Figure 1:**
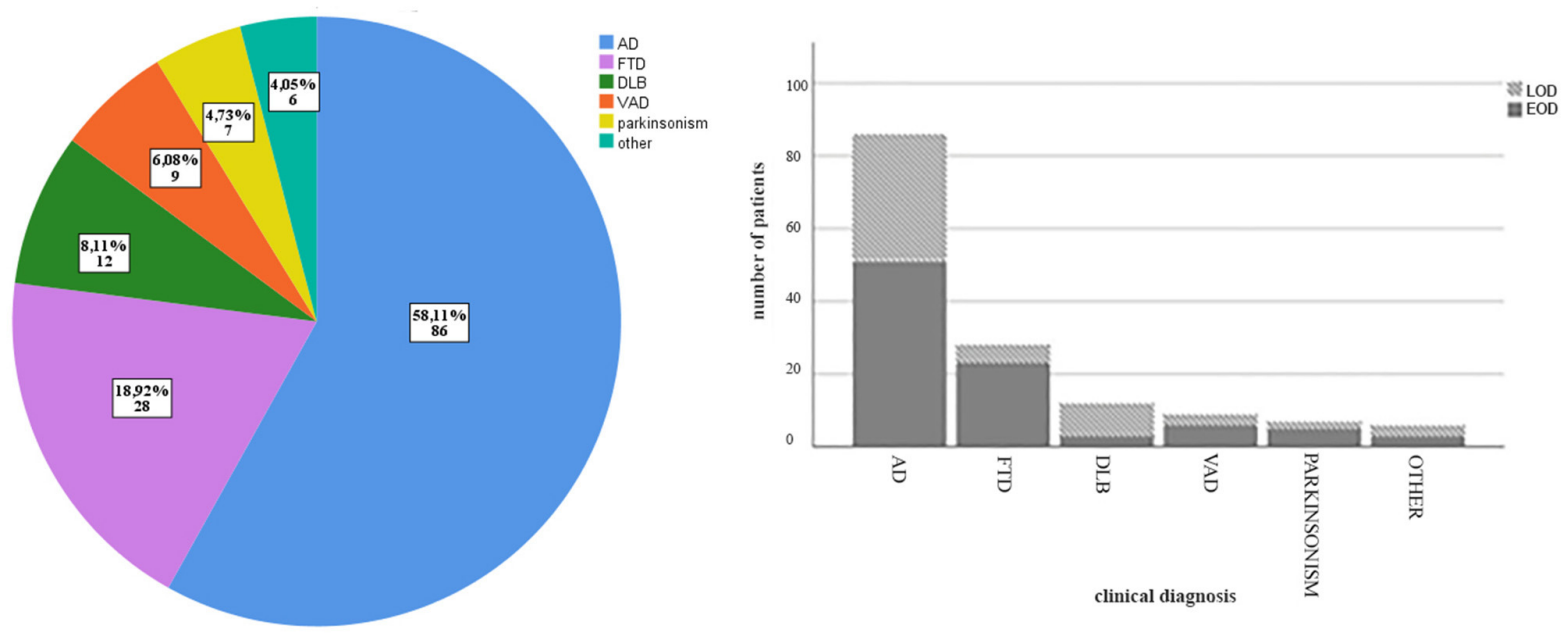
Clinical diagnosis distribution. On the left, clinical diagnosis subgroups in the whole cohort represented by frequency and number of each diagnosis; on the right, clinical diagnosis distribution for EOD and LOD separately.

A total of 148 consecutive eligible patients were included in the present study: 91 EOD and 57 LOD. Figure 1 shows distribution of clinical diagnosis in all group and separately in EOD and LOD. Table 1 shows clinical and demographical features of the recruited patients. Considering the whole cohort, 86 patients were diagnosed as AD (52 EOD, 35 LOD), 28 patients as FTD (23 EOD, 5 LOD), 12 patients with DLB (3 EOD, 9 LOD), 9 as VaD (6 EOD, 3 LOD), 7 patients with ayptical parkinsonism (5 EOD, 2 LOD), and 6 with other dementia (i.e., alcoholic dementia, cerebral amyloid angiopathy; 3 EOD, 3 LOD).

**Table 1:** Clinical and demographical characteristics of patients included in the study.

	**All group (*n* = 148)**	**EOD(*n* = 91)**	**LOD (*n* = 57)**	**Group comparison, P**
Age at recruitment, mean (sd)	70.27 (±8.28)	64.90 (±4.82)	78.56 (±5.02)	*p* < 0.001
Disease duration (years), mean (sd)	5.6 (±3.33)	5.52 (±3.25)	6.25 (±2.74)	0.323
Sex, female (*n*)	86	56	36	0.285
Education (years), median (range)	8 (2–18)	8 (3–18)	8 (2–18)	0.121
MMSE at first visit, mean (sd)	21.92 (±4.54)	21.48 (±5.03)	22.60 (±3.57)	0.149
MMSE at recruitment, mean (sd)	16.51 (±8.42)	15.91 (±8.84)	17.50 (±7.67)	0.376
Early anosognosia (CIRS); median	3	3	4	0.503
Current anosognosia (CIRS), median	6	6	6	0.947
Mean time between first assessment and recruitment (months, sd)	42.68 (±33.42)	36.07 (±30.70)	52.87 (±35.11)	0.003
Current NPI total, median (range)	14 (0–57)	15 (0–54)	14 (0–57)	0.802

*CIRS, Clinical Insight Rating Scale; NPI, Neuropsychiatry Inventory; MMSE, Mini Mental State Examination; sd, standard deviation; ns, not significant*.

Mean age at recruitment was 70.27 (±8.28) years across the whole group, 64.90 (±4.82) years in the EOD group, and 78.56 (±5.02) years in the LOD (*p* < 0.001). Mean disease duration (defined as the time between symptoms onset and recruitment) was 5.6 (±3.33) years in the whole group, with no significant differences (*p* = 0.32) between EOD (mean disease duration 5.52 ± 3.25 years) and LOD (mean disease duration 6.25 ± 2.74 years). There was no difference in sex between EOD and LOD (*p* = 0.28). Similarly, there were no significant differences between EOD and LOD in the MMSE score at the first visit (mean MMSE 21.48 ± 5.03 for EOD, 22.60 ± 3.57 for LOD, *p* = 0.14) or at the moment of recruitment (mean MMSE 15.91 ± 8.84 for EOD, 17.50 ± 7.67 for LOD, *p* = 0.37). At the time of recruitment, MMSE scores were significantly lower relative to the first assessment in both EOD and LOD groups (*p* < 0.001). There were no differences in the relative proportion of AD (*p* = 0.60), VaD (*p* = 0.74), and atypical parkinsonism (*p* = 0.70) between EOD and LOD; on the contrary, FTD diagnosis was more frequent in the EOD group in comparison to LOD (*p* = 0.017), while DLB was more frequent in LOD (*p* = 0.01). Mean time between first visit and recruitment was 42 months, with LOD showing longer time compared to EOD (*p* = 0.003).

Measures of early anosognosia showed that at the first assessment, 83.5% of patients with EOD and 78.9% patients with LOD had some degree of anosognosia [i.e., a CIRS score > 1, as in (25, 26), *p* = 0.484]. Measures of current anosognosia showed that these proportions reached 94.5% of EOD and 98.2% of LOD at the moment of recruitment (*p* = 0.262). Measuring early and current anosognosia as a continuous variable still showed that there were no significant differences between EOD and LOD at either first assessment (mean early CIRS EOD = 3.90, mean early CIRS LOD = 3.61, *p* = 0.50) or time of recruitment (mean current CIRS EOD = 5.64, mean current CIRS LOD = 5.61, *p* = 0.94). Current anosognosia values were significantly higher than values of early anosognosia (*p* < 0.001), even when controlling for the effect of MMSE.

### Association Between Anosognosia and Clinical Diagnosis

**Figure 2:**
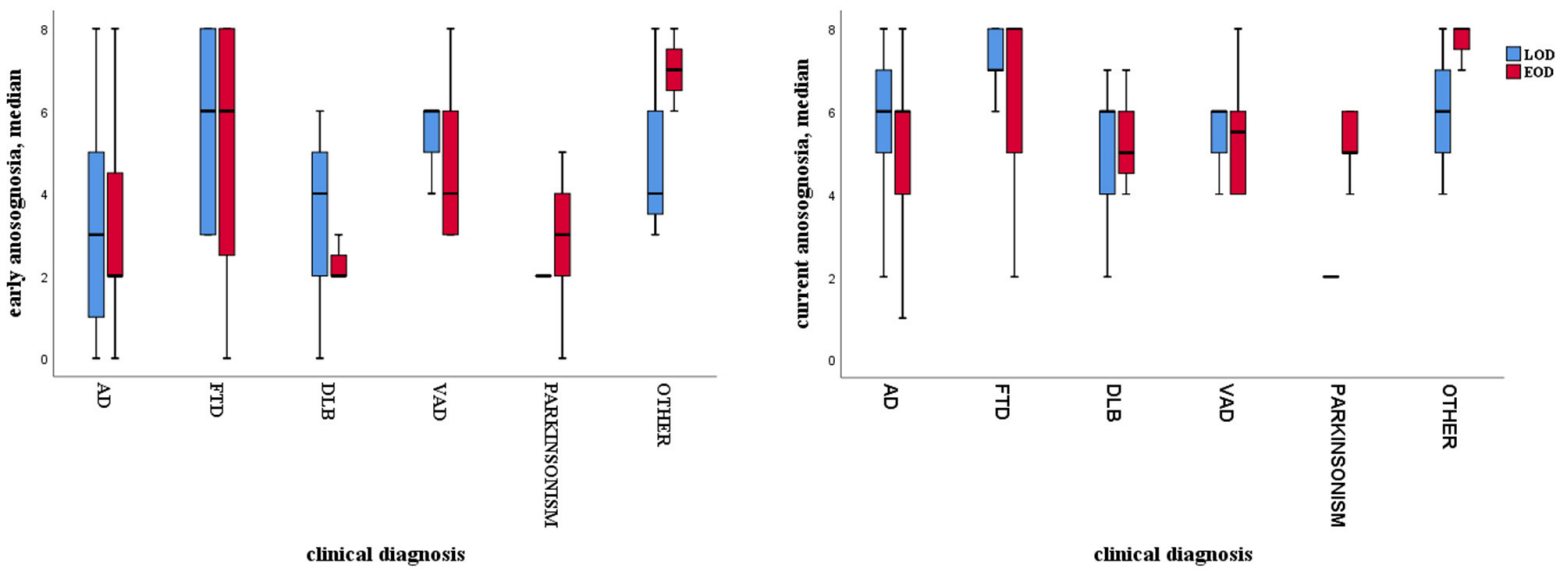
Boxplot of anosognosia level in EOD and LOD for each diagnostic subgroup; on the left, early anosognosia; on the right, current anosognosia.

Comparisons of early anosognosia across different diagnostic subgroups showed that it varied among different clinical subgroups independently of age of onset: FTD patients showed higher mean anosognosia values in comparison to AD patients, in both EOD (mean CIRS FTD-EOD = 5.39, mean AD-EOD = 3.16, *p* < 0.001) and LOD (mean CIRS FTD-LOD = 5.60; AD-LOD = 3.26, *p* < 0.001). The same pattern emerged for current anosognosia, but the differences between FTD and AD patients did not reach significance. Figure 2 graphically depicts early and current anosognosia levels by diagnostic subgroup.

**Figure 3:**
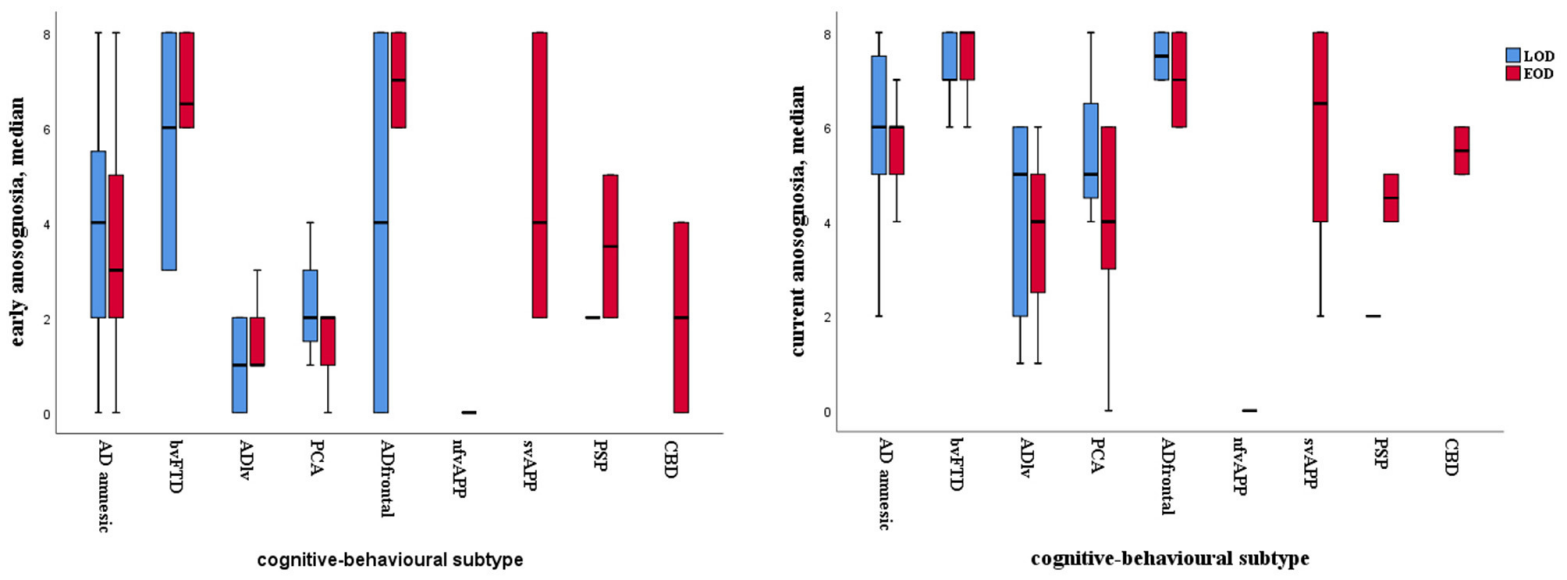
Boxplot of anosognosia level in EOD and LOD for patients classified based on cognitive/behavioral patterns; on the left, early anosognosia; on the right, current anosognosia.

We further subdivided each diagnostic group according to the variant they had presented with, in order to investigate possible differences in level of anosognosia between presentation with predominantly cognitive or behavioral symptoms (12). More precisely, we divided the AD group into amnestic variant, frontal/dysexecutive variant, logopenic variant of primary progressive aphasia, and posterior cortical atrophy. We divided the FTD group into behavioral variant, non-fluent variant of primary progressive aphasia, and semantic variant of primary progressive aphasia. We found that patients with the dysexecutive/frontal variant of AD and those with the behavioral variant of FTD showed highest levels of both early and current anosognosia irrespective of age of dementia onset (Figure 3). More precisely, patients with the behavioral variant of FTD had higher values of anosognosia than those with the amnestic variant of AD (*p* = 0.001), logopenic primary progressive aphasia (*p* < 0.001), posterior cortical atrophy (*p* < 0.001), and non-fluent agrammatic variant of FTD (*p* = 0.013), whereas there were no significant differences between patients with the behavioral variant of FTD compared to those with the frontal/dysexecutive variant of AD.

### Association Between Anosognosia and Neuropsychiatric Symptoms

When studying the presence of behavioral disturbances measured by the NPI, either with its total score or individual subscores for different behavioral symptoms, we found that there was a positive correlation between early anosognosia and current NPI apathy subscore (*r* = 0.267, *p* = 0.001) across all patients and in the EOD group only (*r* = 0.440, *p* < 0.001), but not in the LOD group only (*p* = 0.245). There were no significant correlations between current anosognosia and current NPI total score or subscores, in either EOD and LOD, as well as across all patients.

A linear regression performed in all patients with current total NPI score as the dependent variable and early anosognosia and MMSE as independent variables showed that higher current NPI scores were associated with higher level of early anosognosia (standardized coefficient beta = 0.175, *p* = 0.039, CI 0.047–0.728). When performing the same analysis in EOD and LOD separately, such association remained significant in the EOD group only. When exploring single current NPI subscores in the whole cohort with regression analysis, a significant association between current NPI apathy subscore and early anosognosia was found (standardized coefficient beta = 0.441, *p* = 0.001, CI 0.194–0.688). Such association was specific for the EOD group (standardized coefficient beta = 0.745, *p* < 0.001, CI 0.420–1.071). The same regression analysis performed by adding disease duration, time between first visit and recruitment, and diagnostic subgroup as independent covariates confirmed that current NPI apathy subscore in the EOD group only was associated with early anosognosia (standardized coefficient beta = 0.830, *p* < 0.001, CI 0.353–1.106) and diagnostic subgroup (in particular FTD, VaD, and atypical parkinsonism; standardized coefficient beta = 0.669, *p* = 0.001, CI 0.267–1.071).

## Discussion

In this study, we investigated whether there were differences in the level of anosognosia between individuals with EOD and LOD. We also tested if anosognosia in EOD differs across different types of dementia and their clinical variants, and whether it is associated with neuropsychiatric symptoms.

First, we found that median level of anosognosia was not significantly different between EOD and LOD. Both EOD and LOD reported the same degree of early and current awareness, both when measuring anosognosia as a binary value with CIRS > 1 or when measuring anosognosia as a continuous variable. Only when considering a CIRS score > 0 in comparison to any degree of anosognosia, as previously done by De Carolis et al. (27), was higher percentage of anosognosia found in EOD compared to LOD. To our knowledge, only three previous studies had explicitly investigated differences in anosognosia in EOD and LOD (9–11), reporting conflicting results. Whereas, previous studies had only focused on AD or VaD and did not take into account possible different clinical diagnosis subtypes, in our study, we included different clinical variants of AD as well as of all the types of dementia presenting to dementia clinics. This was especially important considering that non-AD diagnoses are frequent in EOD patients (12).

Second, we found that in both groups, anosognosia increased overtime with disease progression and was higher in FTD patients or, more precisely, in AD and FTD variants associated with involvement of the frontal lobes. More precisely, we found that anosognosia was higher in the frontal/dysexecutive variant of AD and in the behavioral variant of FTD for both early and current anosognosia, irrespectively of age of dementia onset. This finding is consistent with neuroimaging studies showing involvement of frontal cerebral areas in mechanisms of self-awareness, in both healthy subjects (28) and patients (29, 30). Recent cognitive models of anosognosia such as the Cognitive Awareness Model postulated by Agnew and Morris (2) and revised by Morris and Mograbi (31) hypothesized that anosognosia derives from the failure in integrating incoming knowledge with information stored in a personal database, a system thought to be largely dependent on frontal brain structures.

Third, we found a strong association with early anosognosia in the EOD group and neuropsychiatric symptoms, more precisely with apathy, which has been extensively correlated with dysfunction of the frontal lobes, especially the medial prefrontal cortex (32). Higher levels of early anosognosia were positively associated with subsequent apathy, suggesting that patients with lack of awareness at the beginning of their disease are more at risk to manifest apathy and indifference later in the course of the disease. This result was independent from time between first assessment and recruitment and from disease duration. These novel findings are in line with previous cross-sectional and longitudinal studies demonstrating that anosognosia and apathy are strongly associated (33–35) and are both related to frontal lobe dysfunction (36, 37). Among these, the cross-sectional study by Spalletta et al. showed that anosognosia was correlated not only with apathy, but also with the NPI subscores of agitation and aberrant motor behavior in a large cohort of patients with AD. Differently from them, we did not find correlations between anosognosia and other behavioral subdomains beyond apathy. These differences may be due to the fact that we included not only AD but also other dementia diagnoses. Interestingly, the same authors found that anosognosia was not related to NPI scores in the Mild Cognitive Impairment phase, suggesting that apathy only occurs in more advanced phases of diseases. Our longitudinal results add on those of this previous study showing that early anosognosia correlates with subsequent apathy. In their longitudinal study, Starkstein and co-authors hypothesized that the fact that anosognosia predicts subsequent severe apathy could be explained by an asynchrony in frontal lobe damage: whereas anosognosia may arise as an early response to frontal lobe damage, apathy may develop with further frontal involvement along progression of the disease; as alternative, they postulated that patients with anosognosia may fail to search for alternative activities due to their inabilities to recognize their increasing functional limitations and may lose motivation and empathy (35). The fact that association between early anosognosia and apathy was found to be specific for EOD group was somehow unexpected. We speculate that this may be related to the fact that EOD may have greater detrimental effects on global functioning (since it affects people who usually are still actively engaged in work, family and social activities) compared to LOD, thus producing higher degree of demotivation and apathy. Further studies using neuroimaging techniques and deeper neuropsychological investigations will be useful to better understand the different relationship between anosognosia and subsequent apathy in EOD and LOD.

We recognize some limitations for our study. First, we used only a single method for anosognosia assessment, and it is well known that different measures may lead to explore different aspects of awareness (26). Awareness is not an all-or-nothing phenomenon; it may be that patients are aware of some type of impairment but not of others. Whereas measurements based on examiner's ratings usually refer to global and general unawareness (including cognitive, behavioral, and functional abilities), measurements of discrepancy and performance variably refer to different specific domains depending on the questionnaire and neuropsychological tests adopted. All these approaches have significant limitations, and there is no consensus on the most suitable method to determine anosognosia in dementia; nevertheless, a recent review recommended the Clinical Insight Rating scale as the most suitable option, because it is easy and quick to perform in clinical interview and it also has strong psychometric properties (interrater correlation 0.91, internal consistency 0.85) (38). Another limitation is that diagnostic subgroups other than AD and FTD are relatively small, and this may mask additional awareness differences among diagnosis subtype and cognitive pattern. Nevertheless, this is the first study to our knowledge to focus on EOD-LOD awareness differences in multiple diseases and we think that investigating anosognosia on the basis of different neuropathological backgrounds and different clinical variants may provide deeper knowledge about intrinsic mechanism of loss of awareness in dementia. Related to this, it should be noticed that we found that FTD patients showed higher early anosognosia values in comparison to AD patients, in both EOD and LOD groups; this result was somehow expected considering that loss of insight is a prominent clinical manifestation of FTD. For this reason, when exploring the association between anosognosia and neuropsychiatric symptoms, we included the clinical diagnosis as a covariate in the multivariate regression and found that apathy remained significantly associated with early anosognosia in the EOD group independently of clinical diagnosis. Finally, one further limitation of our study is that apathy was not measured by an appropriated and specific scale but only with the NPI item; indeed, the main focus of our study was to investigate the association between anosognosia and neuropsychiatric symptoms, so we decided to include NPI as a global scale for our purpose. Future studies with appropriated scales specific for apathy measure will be useful to better explore the relationship between anosognosia and apathy.

In conclusion, we showed that anosognosia is frequent in EOD, either at the beginning or along the progression of the disease, that it is especially severe in clinical variants characterized by primary involvement of frontal brain regions, and that, in EOD, it is associated with the subsequent development of apathy. These results show that anosognosia should not be underestimated in EOD but rather considered a core important clinical feature. It should be recognized by clinicians and families as early as possible in patients with or at risk of cognitive decline, to allow better guidance and management for the patient and caregiver during the progression of the disease.

## Data Availability Statement

The raw data supporting the conclusions of this article will be made available by the authors, without undue reservation.

## Ethics Statement

The studies involving human participants were reviewed and approved by Comitato Etico dell'Area Vasta Emilia Nord (approval number 186/16). The patients/participants provided their written informed consent to participate in this study.

## Author Contributions

MT: conceptualization, data curation, formal analysis, investigation, methodology, and writing original draft. SS: conceptualization, data curation, investigation, and writing—review and editing. GV: conceptualization, data curation, investigation, and writing—review and editing. CC: investigation, methodology, and writing—review and editing. LF: investigation, methodology, and writing—review and editing. AC: conceptualization, data curation, formal analysis, investigation, methodology, and writing—review and editing. GZ: conceptualization, data curation, formal analysis, investigation, methodology, and writing original draft. All authors contributed to the article and approved the submitted version.

## Conflict of Interest

The authors declare that the research was conducted in the absence of any commercial or financial relationships that could be construed as a potential conflict of interest.
